# Biological and Nutritional Applications of Microalgae

**DOI:** 10.3390/nu17010093

**Published:** 2024-12-29

**Authors:** Sümeyye Sarıtaş, Arda Erkan Kalkan, Kadir Yılmaz, Savas Gurdal, Tolga Göksan, Anna Maria Witkowska, Mauro Lombardo, Sercan Karav

**Affiliations:** 1 Department of Molecular Biology and Genetics, Çanakkale Onsekiz Mart University, 17100 Çanakkale, Türkiye; sumeyyesaritas@stu.comu.edu.tr (S.S.); ardaerkankalkan@gmail.com (A.E.K.); 2 Çanakkale Onsekiz Mart University Rectorate, Çanakkale Onsekiz Mart University, 17100 Çanakkale, Türkiye; kyilmaz@comu.edu.tr; 3 Science and Technology Application and Research Center, Çanakkale Onsekiz Mart University, 17100 Çanakkale, Türkiye; savas.gurdal@comu.edu.tr; 4 Faculty of Marine Sciences and Technology, Çanakkale Onsekiz Mart University, 17100 Çanakkale, Türkiye; tgoksan@comu.edu.tr; 5 Department of Food Biotechnology, Medical University of Bialystok, 15-089 Bialystok, Poland; anna.witkowska@umb.edu.pl; 6 Department for the Promotion of Human Science and Quality of Life, San Raffaele Open University, Via di Val Cannuta, 247, 00166 Rome, Italy; mauro.lombardo@uniroma5.it

**Keywords:** microalgae, functional properties, nutrition, health

## Abstract

Microalgae are photosynthetic microorganisms that have a rapid growth cycle and carbon fixation ability. They have diverse cellular structures, ranging from prokaryotic cyanobacteria to more complex eukaryotic forms, which enable them to thrive in a variety of environments and support biomass production. They utilize both photosynthesis and heterotrophic pathways, indicating their ecological importance and potential for biotechnological applications. Reproducing primarily through asexual means, microalgae have complex cell cycles that are crucial for their growth and ability to adapt to changing conditions. Additionally, microalgae possess bioactive compounds that make them both nutritious and functional. Thanks to their content of proteins, lipids, carbohydrates, vitamins, and minerals, they play an important role in the development of functional food products, particularly by enhancing nutritional content and product quality. Furthermore, studies have demonstrated that algae and algal bioactive compounds support cardiovascular health, immune function, and gut health, especially in relation to obesity and other metabolic diseases. They also contribute to skin health and cognitive functions, including memory. This review article explores the biological, nutritional, and functional properties of microalgae based on the studies conducted.

## 1. Introduction

As photosynthetic microorganisms, microalgae possess the ability to transform sunlight and carbon dioxide (CO_2_) into biomass that is abundant in lipids, proteins, and carbohydrate molecules. They demonstrate notable varity and adaptability to different conditions, growing from hot springs to highly alkaline and hypersaline aquatic environments [1]. The diversity of microalgae provides the cultivation and utilization of various species that have special properties.

**Figure 1:**
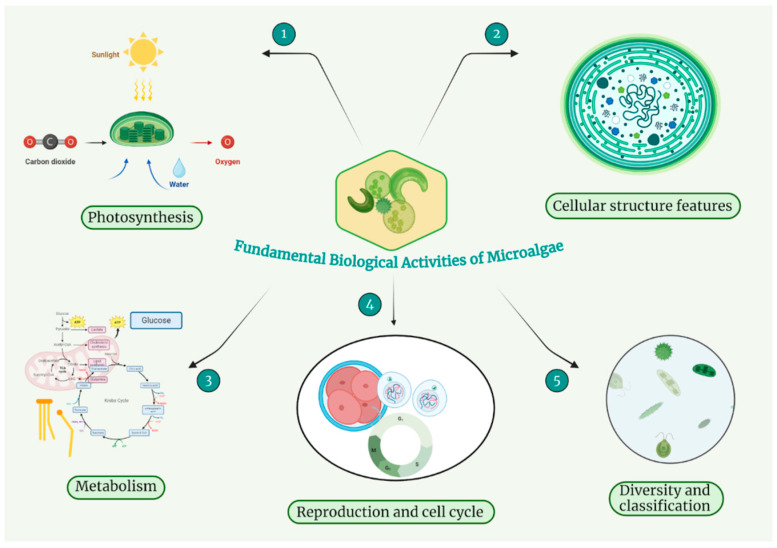
Biological activities of microalgae.

Microalgae, with their rapid growth cycle and carbon fixation ability, demonstrate a new perspective on global issues such as carbon cycling, environmental sustainability, carbon capture, and oxygen production [2,3]. Microalgae cultivation typically involves either open-pond systems or closed photobioreactors [4]. Open ponds are more economical and practical for large-scale operations but are prone to contamination and environmental variations. In contrast, photobioreactors provide a controlled environment with higher productivity, though they come with increased operational costs [4]. They show considerable cellular diversity, ranging from prokaryotic cyanobacteria to complex eukaryotic cells, each adapted to various environmental conditions and contributing to biomass production and ecological resilience (Figure 1) [5,6,7,8]. Their metabolism, primarily photosynthetic but also incorporating heterotrophic pathways, contains complex enzymatic systems for carbon source assimilation and lipid metabolism, highlighting their ecological importance and biotechnological potential [9,10,11,12]. The reproductive strategies and life cycles of microalgae, which include a mix of asexual reproduction and intricate cell cycle processes, are crucial for understanding their growth mechanisms and responses to environmental stressors, including microplastics [5,13,14]. Representing a largely understudied group with potentially up to half a million species, microalgae demonstrate remarkable adaptability and significant ecological and commercial potential, particularly in biofuels and aquaculture, highlighting the urgent need for further research and refined classification methods [15,16].

Microalgae represent both a nutritional and functional alternative to meet the dietary needs of the rapidly increasing human population [17]. Functional foods have become a source of interest for consumers, particularly over the last decade [18,19]. In response to the rising demand and necessity, food companies are utilizing these components to develop alternatives to widely consumed food products or innovative new products, particularly those with enriched components [20]. Algae can be consumed as food products, either fermented or unfermented, and as supplements [21]. In this context, microalgae contribute to the production of functional foods with their rich components [21]. Products supplemented with microalgal biomass exhibit differences in physicochemical properties and biological effects [20]. Furthermore, the addition of microalgae can enhance the macro- and micro-molecule content, thereby improving the nutritional profile of the products [22]. It has been determined by the European Food Safety Authority (EFSA), the US Food and Drug Administration (FDA), and the Turkish Food Codex (TGK) that some microalgae species can be used as supplementary food products and additives [23,24,25]. After these microalgae are evaluated for their nutritional content and functional properties and their food safety is approved, how they will be used is also determined by the relevant institutions [23,26,27,28]. These algae are categorized as novel foods due to their potential roles in sustainable food systems and their promising health-enhancing properties [26,27,28]. The best known of these is *Spirulina*, known for its high protein content [29,30,31]. The rich bioactive content of microalgae has been noted for its potential to reduce the risk of various health issues [32]. It has been demonstrated that they may support metabolic health, cardiovascular health, intestinal health, skin health, and even cognitive function [32].

This review article outlined the general overview of microalgae. To this end, the main features of microalgae as photosynthetic organisms, including rapidly fast growth and carbon fixation, are outlined in detail. In addition, the cellular structures of microalgae and how these structures assist biomass formation are explored. Microalgae are employed in the production of various foods with high nutritional value owing to their unique biological content. These products are classified as functional because of the overall health benefits they provide. The article is exploring these products and the health benefits of microalgae consumption. Additionally, the environmental benefits realized with the carbon fixation capability of microalgae are also considered.

## 2. Biological Activities

Microalgae play an important role in the global carbon cycle and oxygen generation due to their elevated photosynthetic efficiency and adaptability to diverse environments. Their biological processes, including complicated cellular structures, metabolic pathways, and varied reproductive strategies, enable them to survive, reproduce, and thrive in even the most extreme conditions (Table 1).

### 2.1. Photosynthesis 

Photosynthesis is a singular and extraordinary phenomenon of energy conversion, whereby inorganic compounds and light energy are transformed into organic matter by photoautotrophic organisms [58,59]. Both the planet and humanity are intrinsically dependent on photosynthesis for sustaining ecosystems and life as we know it. Although its significance has not been fully appreciated, it is important to recognize that microalgae species possess a crucial and unparalleled ability to transform nearly half of the carbon dioxide present in the Earth’s atmosphere [60]. This carbon dioxide is converted into organic compounds on a yearly basis, primarily through the complex and highly efficient process of photosynthesis, which functions as a vital mechanism for carbon fixation [59,61]. For this reason, it is imperative that greater scientific attention and research efforts be directed toward microalgae as key photosynthetic organisms that play a profound and indispensable role in global carbon cycling and serve as a primary source of production in various ecosystems.

Over the past decade, both researchers and industries have highlighted the potential of microalgae, leading to a growing interest in its various applications [2,3,62]. One standout characteristic of microalgae is its rapid growth rate, which distinguishes it from terrestrial plants. In particular, microalgae’s carbon fixation capability, observed to be 1 to 50 times more efficient than that of land plants, has garnered significant attention around the time [3]. These findings simply show the increasing recognition of microalgae as a greatly sustainable and versatile alternative source for many industrial applications and environmental management.

Microalgae’s ability to perform photosynthesis in both typical and challenging environments makes it one of the most unique and competitive species, compared to many plants and crops that require specific conditions for photosynthesis and efficient carbon dioxide conversion into oxygen [63]. Furthermore, species with optimized, therefore enhanced photosynthetic capacities are among the fastest-growing photosynthetic microorganisms [58]. Another unique feature of microalgae is that it is capable of absorbing dissolved inorganic carbon (DIC) directly from aquatic environments in the form of CO_2_ and HCO^3−,^ a feature not found in terrestrial plants [2,3,62].

Meanwhile, there are some core factors that influence photosynthesis and, therefore, microalgal development. One of these factors is the pH value, which plays a critical role in determining the optimal conditions for microalgal growth [64]. Another key factor influencing the growth of microalgae, with respect to their photosynthetic ability, is the light emission intake from the day/night cycle. It is known that the ideal pH value or range for microalgae can vary significantly from one species to another, which highlights the adaptability and diversity of these organisms in different environments [65,66,67]. Fluctuations in pH may lead to a decrease in photosynthesis rates or, in more extreme cases, result in the complete inhibition of photosynthesis [68].

Salinity is another factor that primarily affects chlorophyll content in microalgae [35]. Low salinity levels have been shown to accelerate photosynthesis by facilitating the uptake of metabolites required for cell growth and development [24]. Temperature is the main factor; for example, a study on *Conticribra weissflogii* (formerly *Thalassiosira weissflogii*) found that as temperature increases, the photon inactivation rate rises, which negatively impacts photosynthetic efficiency [69]. This suggests that higher temperatures may cause a disruption in photosynthesis by increasing the rate at which photons become inactive, affecting both algal growth and photosynthetic output. Numerous other studies have explored the relationship between photosynthesis conditions and microalgae [4,34,35,37].

An important aspect of microalgae is its ability to sequester heavy metals, which allows for the manipulation of nanoparticle production to form in conjunction with chlorophyll [70,71]. Other research has examined how carbon-based nanoparticles can accelerate the light emission spectrum, thereby increasing photosynthesis rates [72,73]. These findings suggest that low concentrations of nanoparticles create an optimal effect, while higher concentrations lead to significant reductions in both growth and photosynthesis.

### 2.2. Cellular Structure 

Microalgae can exist in both prokaryotic forms, such as cyanobacteria, often referred to as blue-green algae. These organisms are distinctive for their ability to move independently while using organic compounds for the carbon capture process [5,6]. On the other hand, eukaryotic microalgae have a more complex cellular structure, consisting of various essential organelles that are crucial for biomass production and the synthesis of other organic compounds [7,8]. These compartments include the nucleus, chloroplast, endoplasmic reticulum, mitochondrion, vacuole, Golgi body, cytoplasm, and cell wall, all of which are critical for the overall function and efficiency of the eukaryotic microalgal cell [7,8].

Microalgae demonstrate a hugely wide diversity in shape, form, and size, with dimensions ranging from approximately 0.5 to 200 μm, as mentioned by Barsanti and Gualtieri (2014) [74]. This variation is not only evident between different genera and species but can also be seen at various stages of growth and development within the same species of microalgae [74]. Additionally, the most abundant morphological forms of microalgae are classified into several distinct categories, including amoeboid, palmelloid (also referred to as capsoid), coccoid, filamentous, flagellate, and, finally, sarcinoid [5].

The morphological shapes and forms of microalgae throughout their lifetime are often a topic of debate, as many microalgal species are not classified within any specific morphological classification group, despite the large number of species present [74]. Consequently, the status of these morphological classifications for microalgae is generally acknowledged as thallus, which can be either unicellular or multicellular. Additionally, they can also be found as stalks, which may form in unicellular, colonial, or multicellular forms [75].

There are various microalgae species and subgenera to be studied to learn more about their structures and characteristic features, such as the green microalga species *Dunaliela salina* species, which is quite famous for many reasons, is a unicellular and part of the Chloropyhta phylum [75]. *Dunaliella salina* is found in a uniform biflagellate form, which helps the species survive harsh conditions such as high levels of induced salinity stress [38,76]. The overall shape of *Dunaliella salina* cells resembles an oval under normal conditions; however, when stressed by osmotic pressure, the shape becomes more spherical [76]. This specific change in shape is thought to be caused by a deficiency in a prominent cell wall and the presence of either a mucous-containing outer layer or a flexible cell membrane [77]. Consequently, it becomes difficult to release the substances within the cells [39]. Meanwhile, another flagellate type, the aflagellate unicellular form, can be abundantly found in diverse conditions and across various phyla such as Cyanophyta, represented by the species *Aphanothece microscopica* Nägeli, as well as in the genus *Porphyridium* of the phylum Rhodophyta [78]. So that, microalgae species carry all these unique characteristics, making it inevitable to consider them a valuable future research topic in many biological and environmental fields, according to these and more related study outcomes.

In terms of colony types of microalgae, amorphous and coenobium are two such types. While coenobium colonies are characterized by more organized cells, often forming a stalk-like structure, amorphous colonies (such as Cyanophyta and Chlorophyta) show no specific arrangement in their cell aggregates [75]. To provide more specific species information, *Chlorella vulgaris*, a well-known species of the Chlorophyta phylum, is famously considered one of the key species in freshwater green microalgae research [40]. The cells of *Chlorella vulgaris* are spherical, with a size range of 2 to 10 μm. They have a cell wall susceptible to enzymatic activity, with a width ranging between 17 and 21 nm [41,42,79]. In addition to these general structural details, the ultrastructural information on microalgae plays an important role in understanding their evolutionary history on Earth. The results of ultrastructural studies on microalgae reveal intriguing data, suggesting nearly 3.6 billion years of evolutionary modifications. This has led to a highly extended phylogenetic tree, with heterogeneous species being abundant and capable of adapting to their environment [80].

### 2.3. Metabolism

Generally, microalgae are organisms that cultivate through photosynthesis, and their metabolism is primarily adapted for this process. However, there are also some heterotrophic microalgae, which, although less common, rely on organic and inorganic metabolites, mainly in dark environments where they do not depend on light sources [81]. Thus, these heterotrophic microalgal species have a highly specialized metabolism for utilizing substances, primarily carbon and lipid sources, while also effectively utilizing other nutrients.

For the metabolic pathway, the cell cytoplasm is important for the compound transfer systems, such as glucose and acetate uptake, which are not independent of the proteins responsible for transduction [48,49,82]. As proton symporters, both *Chlorella vulgaris* and *Ettlia oleoabundans* (formerly *Nannochloropsis oleoabundans*) exhibit glucose intake that is closely related to these proton symporters [83,84]. Another microalgal species, *Galdieria sulphuraria*, can metabolize not only glucose in various ways but also other types of sugars with the help of proton symporters and other transporters—nearly 28 in total [44,45].

Among the metabolites utilized in the metabolism of heterotrophic microalgae, carbon-based metabolites are of overall importance. Related to symporters, glucose is known for its abundance as a carbon-based metabolite in heterotrophic microalgal metabolism, serving as a significant energy storage source compared to other non-carbon and carbon-sourced metabolites [9,10]. The metabolism of glucose is widespread due to its role as a primary substrate in the phosphorylation of glucose-6-phosphate, which is involved in both respiration and cell generation [43,85].

Another crucial carbon-based metabolite in heterotrophic microalgae is glycerol, which is predominantly found in microalgal species located in high salinity environments [86]. Certain algae species, including *Merismopedia quadruplicata* (formerly *Agmenellum quadruplicatum*) and *Nannochloropsis* spp., are capable of metabolizing glycerol in either mixotrophic or heterotrophic environments, promoting growth when conditions such as light or other environmental factors are favorable [52,53]. Interactions with bacteria, such as *Azospirillum brasilense*, can enhance microalgal growth, particularly under stressful conditions like those found in synthetic wastewater media [50,51]. Although glycerol metabolism offers various advantages, our knowledge of its processes under heterotrophic conditions is still limited, particularly in relation to biodiesel production [12].

The assimilation of carbon sources like acetate, glucose, lactate, and glycerol in microalgae involves complex enzymatic systems for transport, activation, and energy generation through pathways such as the oxidative pentose phosphate (PPP) and Embden–Meyerhof–Parnas (EMP) [11,12]. Hexokinases and glucose dehydrogenases play key roles in glucose activation in *Chlorella* and *Euglena* [11]. Glycerol and acetate are metabolized through the glyoxylate and TCA cycles, contributing to ATP and NADH production [12]. The glyoxylate cycle, identified in *Chlorella vulgaris*, requires malate synthase and isocitrate lyase, which are induced under dark, acetate-fed conditions [87]. Additionally, microalgae like *Auxenochlorella pyrenoidosa* (formerly *Chlorella pyrenoidosa)* and *Tetradesmus obliquus* (formerly *Scenedesmus obliquus*) maintain high mitochondrial activity under both light and dark conditions [12,88]. The mitochondrial TCA cycle and electron transport system in microalgae exhibit similarities to those found in higher plants and eukaryotic cells [11].

In heterotrophic microalgae, there are also non-carbon-based metabolites, such as triacylglycerol (TAG), a key lipid metabolite. Its biosynthesis is crucial for primary cell metabolism and is intimately linked to membrane lipid assembly [89,90,91,92,93]. Species such as *Nannochloropsis*, which produce TAG even under nutrient-sufficient conditions, present substantial potential for further investigation [94,95,96]. Progress in molecular techniques, including nuclear transformation and RNA interference, has established *Chlamydomonas* as a model organism for lipid research [97,98,99,100,101]. Algal lipids are classified into two main types: neutral lipids, predominantly triacylglycerols (TAGs) found in cytosolic bodies, and membrane lipids, which include glycolipids in chloroplast membranes and phospholipids in membranes outside the chloroplast [47].

The synthesis of TAG can resemble an electron sink if stress occurs by the photo-oxidative event [102,103]. Most microalgae achieve their highest lipid content in adverse environmental conditions that inhibit rapid growth [104]. These organisms produce several essential polyunsaturated fatty acids (PUFAs), including eicosapentaenoic acid (EPA 20:5 ω-3), docosahexaenoic acid (DHA 22:6 ω-3), α-linolenic acid (ALA 18:3 ω-3), γ-linolenic acid (GLA 18:3 ω-6), and arachidonic acid (ARA 20:6 ω-6) [104]. On the other hand, sterols are primarily found in many eukaryotic organisms and are an integral part of the plasma membrane, which is also quite common in microalgal species [105,106]. It has been observed that extracts from *Schizochytrium* (Bigryra phylum, class Labyrinthulea) have a potent influence on the relationship between intestinal gene expression and cholesterol intake [107]. All of these findings highlight the prevalence of lipid-oriented metabolites in heterotrophic microalgae.

Another important group of metabolites influenced by environmental factors is pigments, including chlorophyll, accessory pigments such as carotenoids, and phycobiliproteins [108,109,110]. For example, *Haematococcus lacustris* (formerly *Haematococcus pluvialis*) produces astaxanthin through autotrophic processes, whereas *Chromochloris zofingiensis* (formerly *Chlorella zofingiensis*) generates higher yields under heterotrophic conditions [111]. Additionally, phycocyanin is found in Cyanobacteria like *Limnospira platensis* (formerly *Spirulina platensis*) and in microalgae such as *Galdieria sulphuraria*, which can produce this pigment in heterotrophic cultures with sufficient nitrogen but limited carbon sources [112,113]. Thus, microalgae species are not only containing the majör micronutrients but also possessing distinct pigments that play various important roles in their biological processes and that can be manipulated through extended biotechnological applications.

Moreover, beyond these main compounds, it is globally recognized that microalgae make use of other vital substances in their metabolism mechanisms. Each of the elements discussed in this section plays a critical role in many metabolic pathways, significantly contributing to the expansion and development of microalgal species.

### 2.4. Reproduction and Life Cycle

The family of algae usually shares similar reproductive characteristics with other eukaryotic organisms, such as the generation of a zygote through the merging of a sperm and egg cell; however, this is not always the case for every genus of algae [5]. For example, species like *Chlorella* and *Nannochloropsis* could rely more on asexual reproduction rather than sexual reproduction, and this preference is still not fully explored to date. There are many thoughts about this case, but its relation mainly associated with the preference is rooted in either deficiencies in sexual variability or challenges related to mating and finding a suitable partner [5]. While the topic of the cell cycle in algae has been a highly major area of study for several decades, these studies have largely focused on key models such as *Desmodesmus* [114]. The efforts and findings in this domain have been crucial for gaining a deeper understanding of the mechanisms behind the cell cycle of microalgae, paving the way for even more detailed future research in this field.

The cell cycle in microalgae is a complicated process that consists of various important stages designed to replicate cellular structures. These stages include growth, DNA replication, nuclear division, and cellular division, also referred to as protoplast fission [13]. For the purpose of this review, the cycle can be divided into two main phases: pre-commitment and post-commitment [13]. The commitment point plays a pivotal role; at this stage, a cell that has achieved a certain size and content is ready to proceed through the cell cycle without the need for additional growth. This indicates a prominent level indicator for the process of cell cycle maintenance. Once past this point, the cell enters the DNA replication-division sequence, which involves DNA replication, mitosis, and cytokinesis. While the timing of these core stages is relatively well understood, the preparatory phases, especially those related to the accumulation of essential molecules for DNA replication, remain less clearly defined [13].

### 2.5. Diversity and Classification

Over the time, the diversity of microalgae has significantly expanded, with estimates suggesting the existence of approximately half a million species, but only a fraction of these, around 40,000, have been thoroughly described and studied in detail [16]. While the morphological and pigment-based methods were once the primary means of classification, advances in molecular biology have allowed for a more refined understanding of this vast and still largely unexplored group of organisms [15]. This highlights the considerable biodiversity within microalgae and the need for further research to fully catalog and comprehend the ecological roles and potential applications of the remaining, undescribed species. 

Microalgal species exhibit broad diversity in their genetic and phenotypic characteristics, allowing them to thrive in nearly all environments where plants can grow, including freshwater, seawater, and even extremely saline aquatic environments like the Dead Sea. Microalgae can also be found in various locations, ranging from urban city walls to extremely hot desert biotic crusts [56,115,116]. Antarctic snow and high-altitude mountain region [117,118]. Their size can range from 5 µm to 50 µm, and they can exist as unicellular organisms or in other forms with various colors. Additionally, it has been reported that microalgae can be isolated independent microorganisms or sometimes form relationships with other organisms, such as lichens [119].

The hierarchical classification of life existed before the concept of evolution, which has since made it difficult to categorize certain organisms, especially simpler forms like Chromista kingdom, microalgae, and brown seaweeds (Phaeophyceae). The Kingdom proposed in 1986 by J. Hogg and Ernest Haeckel, which included all organisms that did not have “complex tissues”, was called Protoctista (or Protista) and included a great diversity of organisms, such as algae, fungi, and sponges.

A better, but not perfect, solution was proposed in 1956 [120], when Copeland proposed the Kingdom Monera to include prokaryotic single-celled organisms. Copeland’s 4-Kingdom classification system was considered, at the time, a “step forward”, but because in its elaboration it did not take into account different types of nutrition, several classification problems arose, particularly with some organisms included in the Kingdom Protista. Work from the end of the 1990s demonstrated that organisms called “Prokaryotes” had greater diversity than previously thought. The discovery of a third cell type (Archeobacteria) was made by C. R. Woese and G. E. Fox [121,122]. Molecular genetic work carried out in the 90s revealed that anaerobic bacteria found in conditions called “unsuitable for life” were genetically and metabolically different from the others. These bacteria, called archaebacteria, or simply Archaea, were considered “living fossils” because they survived in places with environmental characteristics similar to those found on Primitive Earth (without oxygen). DNA and RNA analyses showed that instead of the 5 Kingdoms there were, in fact, 3 Domains: Archaea, Bacteria, and Eukarya, and several Kingdoms [121]. The original three-kingdom system expanded to include more kingdoms like Eubacteria, Archaea, Animalia, Plantae, Fungi, and Protozoa [123]. Molecular data made classification even more complex, especially with groups like Chromista, whose members (e.g., brown algae, diatoms) originated from secondary endosymbiosis with red algae. Despite numerous reclassification efforts, no system has fully resolved the placement of algae and protists [124]. The intricate relationships caused by plastid evolution and gene transfer make it hard to trace their evolutionary history. As a result, many argue against rigid hierarchical classifications and advocate for flexible groupings that can adapt as new information becomes available [125].

The phylasa being Chlorophyta, Rhodophyta, Haptophyta, Stramenopiles, and Dinoflagellata encompass diverse microalgal genera used in commercial applications. Chlorophyta members like *Chlorella vulgaris*, *Dunaliella salina*, and *Haematococcus lacustris* are notable for lipid, hydrocarbon, and astaxanthin production, while *Botryococcus braunii* shows promise for biofuel generation [126]. Rhodophyta species, such as *Porphyridium cruentum* and *Dixoniella grisea* (formerly *Rhodella reticulata*), are valued for their phycobiliproteins [127]. Haptophytes like *Tisochrysis lutea* and *Rebecca salina* (formerly *Pavlova salina*) are widely used in aquaculture due to their nutritional value [128,129]. Stramenopiles, including *Nannochloropsis*, and diatoms like *Skeletonema costatum* and *Thalassiosira pseudonana* are key for live feed in aquaculture [130,131]. Dinoflagellata, represented by *Crypthecodinium cohnii*, is utilized for docosahexaenoic acid production [132]. In total, it is clear that the mentioned microalgae genera and species display significant potential for commercial applications aimed at meeting economic demands across sectors ranging from biofuels to aquaculture and nutritional products for the food and supplement industries.

In summary, microalgae represent a vastly diverse and still largely unexplored group of organisms, with estimates suggesting there could be as many as half a million species. They thrive in a wide range of environments, demonstrating impressive adaptability. Although there have been historical challenges in classifying these organisms, recent advances in molecular techniques are quite important in their ecological roles. Microalgae hold significant promise for various applications, particularly in areas like biofuels and aquaculture, highlighting the need for ongoing research in this field.

## 3. Nutritional Aspects 

It is widely known that microalgae exhibit high nutritional value as they are very rich in crucial molecules, including lipids, proteins, and carbohydrates [133]. When evaluating the macronutrient composition of microalgae, including proteins, carbohydrates, and lipids, it is evident that they are a nutrient-dense source [134]. Although the composition of these components varies among different species of microalgae, studies have consistently demonstrated their richness in macronutrients [135]. 

Since the 20th century, microalgae have been utilized as an alternative protein source, mainly due to their high protein and complete amino acid contents [29,136]. It is widely known that the human body lacks the ability to synthesize certain crucial amino acids. Given the elevated concentration of essential amino acids, microalgae emerge as a promising alternative protein source for human consumption. *Limnospira platensis* (formerly *Spirulina platensis*), a filamentous cyanobacterial species, has been extensively employed as an alternative dietary supplement owing to its elevated protein content and nutritional significance [30,137]. 

Furthermore, microalgae serve as a notable source of carbohydrates, existing within both the cytosols and chloroplasts in various forms of polysaccharides [138]. Microalgae consists predominantly of polar lipids, encompassing phospholipids and glycolipids, along with monoglycerides (MAGs), diglycerides (DAGs), and triglycerides (TAGs). Additionally, they exhibit a diverse array of free fatty acids, including polyunsaturated fatty acids (PUFAs) such as omega-3 and omega-6 [139,140].

It is well documented that microalgae contain important vitamins such as vitamins A and B, as well as minerals like potassium, iron, magnesium, etc. [141]. Components including β-carotene, astaxanthin, polyphenols, and phycobiliproteins are categorized as micronutrients [20]. Certain microalgae species are renowned for containing phenolic compounds known for their antioxidant activity [142,143]. Microalgae are a good source of complex compounds known as pigments, including zeaxanthin, lutein, and beta-carotene, a sub-class of hydrocarbons [144,145]. The main commercial microalgal species are *Limnospira platensis* (formerly *Spirulina platensis*), *Chlorella vulgaris*, *Dunaliella salina*, and *Haematococcus lacustris* (formerly *Haematococcus pluvialis*). The production and usage of these species are important for different industries, including food, supplements, cosmetics, and feed. The reason why they are crucial is that they are composed of contents, mainly rich in protein, carbohydrates, and lipids (Table 2) [31,51,79,146,147]. 

### 3.1. Microalgae in the Food İndustry

Microalgae have attracted consumers’ attention due to their rich nutritional values and valuable bioactive compounds [27,151].

In general, microalgae have enriched various food products such as bread, pasta, kefir, and ice cream by being incorporated into these products (Table 3). At this point, the most commonly used and notable microalga is *Limnospira platensis*, a blue-green algal species with a high nutritional value [152]. This alga is rich in both protein and essential fatty acids, and studies have been conducted indicating that it reduces the risk of developing various diseases [152,153].

The use of algae in the production of fermented foods has become increasingly common [21]. In this context, a study focused on enriching soy milk-based kefir using *Haematococcus lacustris* microalgae was recently conducted by Tavşanlı et al. [154]. The nutritional and microbiological properties of the products were evaluated with the addition of microalgae in different ratios. According to this study, the addition of microalgae resulted in an increase in the protein content of the products while causing a decrease in lipid content. Additionally, it was determined that the addition of microalgae increased the microbial diversity in the products.

The increasing consumption of sauces has raised the question of how healthier sauces can be produced. In this context, the use of probiotic yogurt in sauce production has become more common [155]. In a study, probiotic yogurt sauce was added with *Limnospira platensis* (formerly *Spirulina platensis*), and it was found that this enriched microalga increased the viability rate of the probiotic bacteria.

In a study carried out by Hernández-López et al., the addition of *Nannochloropsis* sp. and *Tetraselmis* sp. microalgae during the production of wheat tortillas was evaluated in terms of their physicochemical properties and macromolecular component content [20]. It was revealed that as the concentration of microalgae increased, the color of the product darkened, which was attributed to the high pigment content of these two microalgae species, particularly chlorophylls and carotenoids. Tortillas containing *Nannochloropsis* sp. exhibited higher chromatic values and, correspondingly, a more intense green color than others. However, when comparing the physicochemical analyses of control samples and those containing microalgal biomass, similar water activity, pH, and moisture content values were determined, indicating that the addition of microalgal biomass did not have a significant effect on the shelf life of the product. Moreover, the added microalgae were capable of enhancing the macromolecular content. The incorporation of *Nannochloropsis* sp. and *Tetraselmis* sp. microalgae into wheat tortillas improved the protein, fiber, lipid, vitamin, and mineral content of the products, thereby increasing their nutritional value. All products containing microalgal biomass were found to be richer in protein, ash, and lipid content compared to the control. Conversely, the carbohydrate content was higher in the control sample. Furthermore, a significant increase in total phenolic and total carotenoid content was observed with the addition of microalgal biomass. Changes in these contents were associated with antioxidant properties, and both DPPH (1,1-diphenyl-2-picrylhydrazyl) and FRAP (Ferric Reducing Antioxidant Power) assays indicated a proportional enhancement in antioxidant capacity. According to the sensory analysis results, microalgae-enriched tortillas were positively evaluated by consumers in terms of taste, aroma, and overall acceptance.

Ice cream, a product that appeals to consumers of all ages, can have its nutritional and product quality enhanced through the addition of microalgae [22,156]. A study conducted at this point has found that the sensory properties of the products improve with the incorporation of microalgae as a food component in ice cream [22]. It has been emphasized that the type and concentration of the added microalgae directly affect the color of the ice cream. In a similar manner, the addition of *Spirulina platensis* has enriched the product quality, sensory, and rheological properties of the ice cream [156].

Overall, as the nutritional properties of the product improve with the added microalgae, both the rheological and sensory characteristics also change [157]. This is related to the biomass and amount of the added microalgae.

### 3.2. Novel Foods and Regulatory Aspects of Microalgae

**Figure 2:**
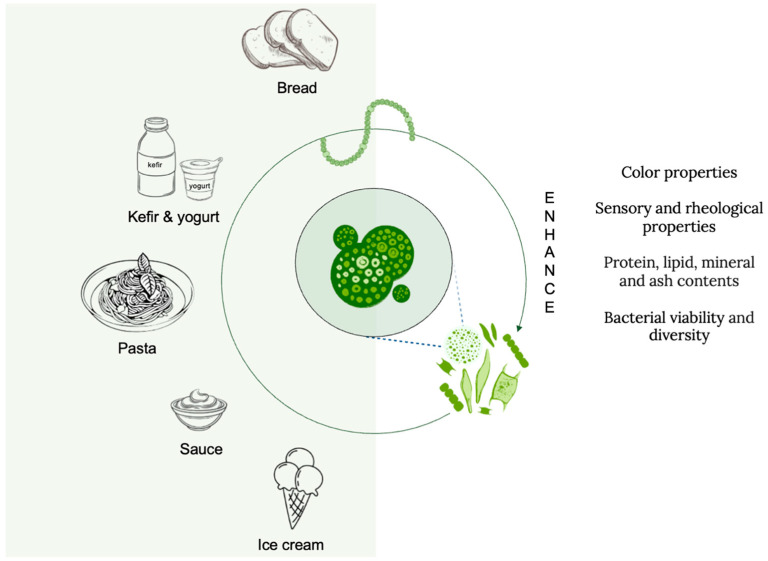
Microalgae-enhanced food products.

Microalgae biomass, which is considered novel foods by EFSA, is increasingly used in the production of functional foods [28,158]. Functional foods have an impact on health beyond their functional value [18,159]. This has enabled the food industry to utilize microalgae as an innovative biological resource by incorporating them into new products (Figure 2) [21]. FDA in the United States, Novel Food Regulation (EU) and EFSA in Europe, and TFC in Türkiye have indicated some species are safe for use as food and supplements [26,160]. The FDA has indicated *Spirulina* and *Chlorella* to be nutritionally safe; the use of microalgae rich in nutrients as food supplements and food colorants has been classified as generally recognized as safe (GRAS) [26,161]. Additionally, the EU and EFSA have classified microalgae as innovative foods and have approved their use in dietary supplements, food, and beverages, as well as protein powders. In Türkiye, the use of microalgae as a food additive and colorant and as a food supplement is permitted by TFC [162]. All these laws are skeptical about the consumption of new species and indicate that a series of analyses and evaluations should be carried out [160,161,162].

The use of microalgae as nutritional ingredients requires certain conditions [162]. These conditions are set to ensure the safety of foods in terms of toxins, heavy metals, coagulants, and microbial risks or allergenic reactions [160,163]. Due to these risks, the use of microalgae species that will not cause a problem in terms of food safety is permitted by the FDA, EFSA, and TFC after completing some tests, but not all microalgae. Before using these microalgae species, a series of analyses are carried out to determine whether they comply with the standards [160,162]. For this reason, research focuses on the requirements of microalgae with rich nutritional content in terms of food safety [24,162,163].

One of the microalgae considered a novel food is *Schizochytrium* sp., thanks to its high DHA content [164]. This evaluation is carried out after the safety of microalgae for consumption is approved. With this feature, *Schizochytrium* sp. provides a sustainable alternative to fish oils. Another example is *Chlorella vulgaris*, thanks to its protein content and functional properties [165]. This algae, considered a nutritious food source, is rich in macro and micro components, including essential amino acids, omega-3 polyunsaturated fatty acids, minerals, and vitamins. These compounds have been linked to potential benefits for cardiovascular health, immune function, and modulation of the gut microbiota [105,149,166,167]. After being approved for its safety, it has begun to be used as a food supplement and food additive. The mentioned microalgae are generally available as liquid suspensions and fractionated components such as protein isolates or lipid extracts [168]. The most common commercial species, *Spirulina* and *Chlorella*, are available in dried form on the market and are made into capsules and tablets for use as supplements [30,168]. *Schizochytrium* is evaluated as an infant formula and fish oil alternative and is available in lipid extract, fractionated components, and dry form [164].

The inclusion of microalgae in functional foods is an innovative approach to improve nutritional profiles and promote health benefits. Products such as bread, pasta, and dairy alternatives enriched with microalgal biomass have shown improved sensory, rheological, and nutritional properties. The integration of *Tetraselmis chui* in tortillas increased protein, lipid, and ash content while improving antioxidant capacity [20]. *Haematococcus lacustris* has been incorporated into soymilk kefir to improve protein content and microbial diversity [154]. Ice cream formulations enriched with *Spirulina platensis* have demonstrated improved sensory acceptability and nutritional value [22,156].

Despite the growing interest in microalgae as novel foods, challenges remain. These include optimizing large-scale cultivation methods, ensuring product safety by minimizing contamination risks, and overcoming consumer acceptance issues related to taste and texture. Continuous research and innovation are essential to expand the applications of microalgae in sustainable food systems. In summary, the classification of microalgae as novel foods by regulatory authorities underscores their potential to address nutritional and environmental challenges. Their incorporation into functional foods represents a promising avenue to improve public health and promote sustainable dietary practices.

### 3.3. Health Benefits Associated with the Consumption of Microalgae

The functional properties of algae mentioned are supported by their effects on health (Table 4). The classification of algae as a sustainable and nutritious alternative protein source also encourages its consumption to improve health markers [169,170]. Metabolic diseases are associated with risk factors such as insulin resistance and obesity [171]. These conditions are linked to a range of diseases that globally affect human health, including cardiovascular diseases, diabetes, and obesity [172,173]. Clinical studies are attempting to determine the effects of microalgae consumption on metabolic health [174]. In a study conducted by García et al., the effects of 60 days of microalgae supplementation on the hematological, anthropometric, biochemical, and hormonal parameters of healthy men were evaluated [171]. Participants received *Tetraselmis chui* microalgae supplementation in two different doses, 25 mg and 200 mg. The groups receiving the microalgae supplementation showed an increase in lean mass and a reduction in body fat percentage. Additionally, enhancements were observed in hematological parameters and hormone levels. *Tetraselmis chui* supplementation has a positive effect on blood lipid parameters, especially high-density lipoprotein levels. It has been stated in the study that it may have effects on reducing dyslipidemia with increasing levels. These effects have also been added to dyslipidemia thanks to the components such as fiber, phytosterols, and long-chain omega-3 fatty acids contained in *Tetraselmis chui*. In addition, *Tetraselmis chui* supplementation may take part in regulating the levels of some cytokines that are involved in regulating the inflammatory response in the body. According to the results of the study, it was determined that it may play a role in improving inflammation by reducing the levels of the pro-inflammatory cytokine known as Tumor Necrosis Factor-alpha and in reducing inflammation by increasing the levels of the anti-inflammatory cytokine called Interleukin-10.

Similarly, in a study aimed at investigating the potential effects of incorporating *Nannochloropsis oceanica* into the diet on metabolic syndrome, a rise in lean mass and a reduction in fat mass were observed [175]. These results are thought to be due to the essential amino acids, proteins, high PUFA, carotenoids, and vitamins contained in *Nannochloropsis oceanica*. It has been shown that this alga can provide changes in the intestinal microbiota, thanks to its rich nutritional elements.

In a study, the effects of the addition of *Diacronema lutheri* microalgae on the metabolic health of Wistar rats fed a high-fat diet were analyzed [176]. This addition resulted in improvements in plasma glucose and insulin levels. Additionally, it demonstrated the diverse effects of supplementation with *Diacronema lutheri* on the regulation of inflammatory cytokine levels. Moreover, a high-fat diet led to a significant increase in liver triacylglyceride and total cholesterol levels in Wistar rats. *Diacronema lutheri* supplementation significantly reduced this increase caused by a high-fat diet. The supplement provided a 74% reduction in triacylglyceride levels and a 39% improvement in total cholesterol levels. The findings of the study suggest that *Diacronema lutheri* supplementation may help alleviate liver lipid metabolism disorders induced by diet.

**Table 4:** Health benefits associated with the consumption of microalgae.

Health Benefits	Microalgae	Effects	References
Metabolic health	*Chlorella vulgaris* (Chlorophyta)	-Enhance taste score -Influence glucose homeostasis	[169]
	*Phaeodactylum tricornutum* (Bacillariophycea)	-Influence glucose and insulin homeostasis	[177]
	*Tetraselmis chui* (Chlorophyta)	-Exhibit antioxidant, and anti-inflammatory effects	[173]
	*Diacronema lutheri* (Pavlovophyceae)	-Influence glucose and insulin homeostasis	[176]
	*Tetraselmis chui* (Chlorophyta)	-Influence hematological and hormonal parameters	[178]
	*Chlorella vulgaris* (Chlorophyta) *Chlorococcum amblystomatis*	-Exhibit lipid-reducing activity and anti-inflammatory activity	[172]
	*Nannochloropsis oceanica*	-Exhibit lipid-reducing activity -Influence gut microbiota	[175]
	*Tetraselmis chui* (Chlorophyta)	-Influence hematological parameters	[174]
	*Tisochrysis lutea* (Coccolithophyceae)	-Influence cholesterol levels -Exhibit anti-inflamatory effects	[171]
Cardiovascular health	*Auxenochlorella pyrenoidosa* (Chlorophyta) *Microchloropsis salina* (Eustigmatophyceae)	-Influence cholesterol levels -Influence vitamin and mineral levels	[179]
	*Limnospira platensis* (Cyanobacteria)	-Exhibit antihypertensive activity	[180]
	*Nannochloropsis* sp. (Eustigmato-phyceae)	-Influence cholesterol levels	[181]
	*Schizochytrium sp.* (Thraustochytriaceae)	-Influence cholesterol levels	[166]
Immune health	*Euglena gracilis* (Euglenophyta)	-Influence cholesterol levels -Exhibit natural killer cell activity	[167]
Gut health	*Phaeodactylum tricornutum* (Bacillariophyceae)	-Influence SCFA levels -Influence microbial diversity	[182]
		-Influence cholesterol levels	[17]
	*Limnospira maxima Chlorella vulgaris* (Chlorophyta)	-Influence incidence of diarrhea	[183]
Skincare	*Dunaliella salina* (Chlorophyta)	-Exhibit antiglycation and anti-Inflammatory activities	[184]
	*Scenedesmus rubescens* (Chlorophyta)	-Modulate hyperpigmentation	[185]
Cognitive function	*Phaeodactylum tricornutum* (Bacillariophyceae)	-Influence mood state	[186]
	*Limnospira maxima* (Cyanobacteria)	-Influence visual learning and visual working memory	[187]
	*Euglena gracilis* (Euglenophyta)	-Influence sleep quality -Influence the autonomic nervous system	[188]
		-Influence oxidative stress -Influence the autonomic nervous system	[170]

In view of the increasing weight gain and obesity issues associated with the pandemic, it highlights the significance of both nutrition and exercise in the weight loss process [177]. A recent study that outlined the role of microalgae supplementation in this process revealed improvements in glucose metabolism and insulin sensitivity parameters among obese women [177]. Collectively, weight and fat loss were also observed. Additionally, while there was no significant difference in exercise volume, it was determined that the microalgae supplementation group engaged in more physical activity compared to baseline.

The inclusion of microalgae in the diet has been shown to impact cardiovascular parameters [180,181]. In a recent study conducted by Sandgruber et al., the microalgal *Auxenochlorella pyrenoidosa* (Chlorophyta) and *Microchloropsis salina* (Eustigmatophyceae), known for their rich nutritional content, were investigated for their effects on human health, particularly cardiovascular health, through regular consumption [179]. Improvements were observed in vitamin D, C, and B12 levels, as well as in the fatty acid profile. A significant reduction in cholesterol levels was detected. Additionally, decreases in glycated hemoglobin (HbA1c) levels were observed, which was associated with a reduced risk of diabetes. Additionally, microalgae are capable of producing bioactive compounds that support immune function. Similarly, the study aimed to analyze how DHA affects fatty acid profiles [166]. Consumption of DHA, which is obtained from Schizochytrium sp., has been associated with enhancement of HDL and total cholesterol.

A study investigated the effects of *Euglena gracilis* consumption on immune function in participants with high levels of perceived stress [167]. The results showed that consumption of *Euglena gracilis* provided a significant reduction in cholesterol levels compared to the control group. Similarly, while low-density lipoprotein cholesterol increased in the control group, it decreased in the *Euglena gracilis* group. However, there were no significant differences in high-density lipoprotein cholesterol between the groups. Additionally, the *Euglena gracilis*-consumed group demonstrated a notable increase in natural killer (NK) cell activity in 8 weeks. According to the cortisol and stress evaluations performed in the study, it was determined that the activities of NK cells increased significantly at the end of 8 weeks.

Intestinal health is associated with many factors that influence human health. Microalgae play important roles in gut health due to their rich composition of components that can exhibit antioxidant and anti-inflammatory effects [17,183]. A study conducted in 2022 investigated the effects of the microalga *Phaeodactylum tricornutum* on gut health [182]. In this study, conducted on mice, it was found that microalga diets increased the production of short-chain fatty acids (SCFAs), including acetic acid, butyric acid, and propionic acid. When inflammatory markers were evaluated, an increase in tumor necrosis factor-α levels was observed. This suggested that microalgae may lead to intestinal inflammatory responses. On the other hand, microalgae consumption was associated with some changes in intestinal taxonomy. In particular, a significant decrease in the ratio of Firmicutes and Bacteroidota bacteria is observed, indicating possible changes in the gut microbiome.

Skin health is subject to both intrinsic and extrinsic aging processes [184,185]. In this context, a recent study investigated the effects of *Dunaliella salina*, which is capable of producing high levels of β-carotene and its precursor carotenoids, on the skin [184]. According to the results, the extract obtained from *Dunaliella salina* exhibited significant antiglycation and anti-inflammatory effects, demonstrating its potential to prevent glycation-induced skin damage. According to the results, the extract obtained from *Dunaliella salina* exhibited significant antiglycation and anti-inflammatory effects, demonstrating its potential to prevent glycation-induced skin damage. Ex vivo studies were conducted to evaluate the antiglycation effect of *Dunaliella salina* extract. According to the results of these studies, it was determined that *Dunaliella salina* extract exhibited a strong antiglycation effect on human skin explants. At the same time, it was determined that the anti-inflammatory effect of *Dunaliella salina* extract caused a decrease in the levels of interleukin 6 and 8 in 24 h treatment, while it increased the levels of the main antioxidant and anti-inflammatory marker called nuclear factor erythroid 2 by 19%. In addition, studies to determine the protective effects under intense UV radiation conducted with 25 female participants determined that there was a decrease in glycation scores, red spot number and area, and wrinkle number and volume.

The use of products that support cognitive functions is common in studies aimed at improving cognitive development and increasing focus [188]. Particularly, gamers who wish to maintain high performance often consume caffeinated beverages and energy drinks [186]. A recent study investigated how the combination of *Phaeodactylum tricornutum* microalgae extract with guarana affects cognitive functions [186]. Despite the positive effects on cognitive functions, it did not significantly impact game performance. It was determined that the high-dose supplementation led to fewer erroneous responses in cognitive function assessments, faster reaction times, and increased light reaction sensitivity. Additionally, it was found that the group receiving high-dose supplementation demonstrated a better quality of life. In another study, the aim was to investigate how cognitive impairment and the diseases it may cause change following supplementation with *Limnospira maxima* extract in elderly individuals [187]. This supplementation was found to lead to improvements in visual learning and visual working memory tests. Additionally, while not significant, an increase in overall antioxidant capacity was also observed.

In summary, the consumption of microalgae has a range of health benefits; however, the advantages and safety of consumption have not been adequately elucidated, thus necessitating further clinical studies.

## 4. Environmental Effects 

Microalgae are suggested as a remedy for various challenges faced by humanity, spanning the realms of food, supplements, medicine, biofuels, etc. Besides, the applications of microalgae extend to addressing environmental concerns, including seawater modification, wastewater treatment, and mitigating greenhouse gas emissions [189]. The broad spectrum of environmental benefits associated with microalgae highlights their potential to contribute to sustainable solutions. Initially, microalgae play a fundamental role in treating wastewater environments through a process known as phycoremediation, presenting a potential alternative for commercial procedures [190]. To purify wastewater in diverse power plants and mining environments, microalgae exhibit substantial effects in absorbing heavy metals as a sorbent [191]. They contribute to optimizing the acidity of wastewater by eliminating, absorbing, or precipitating heavy metals such as Cr, Zn, and others. Consequently, the realkalizing activity of microalgae can be seen in the studies [192]. In addition, microalgae species are used for the nitrogen recovery role for efficiently reuptaking the nitrogen from rich mineral-source wastewater [193]. Additionally, it has been observed that the growth of algae possesses the potential to enhance the auto-depuration effects by releasing oxygen [194]. The same effect can be seen in a technique, namely greenwater, widely applied in marine fish hatcheries, and the availability of oxygen is guaranteed in larvae tanks as a result of the photosynthesis by the use of the live microalgae. Furthermore, as previously noted, microalgae can serve as biofuel sources, and during their cultivation, it has been observed that they exhibit CO_2_ fixation activity from the flue gas [195]. The significant contributions of microalgae, including greenhouse gas emission reduction, heavy metal removal, and nitrogen recovery, emphasized its importance in environmental improvement applications. The important impacts of microalgae on the environment make them a perfect candidate for comprehensive environmental reclamation efforts, complemented by their substantial influence on various industries. 

## 5. Conclusions

To sum up, microalgae species play a crucial role in sustaining life on Earth through their remarkable capability for photosynthesis, which involves the conversion of inorganic substances and light energy into organic compounds. This process not only assists in global carbon cycling but also contributes to oxygen generation, further demonstrating their environmental sustainability. Microalgae possess distinct and diverse cellular structures, ranging from some of the simplest prokaryotic forms to more complex eukaryotic organisms, allowing them to adapt to vastly different environments and ecosystems, making them ecologically resilient organisms.

Microalgae demonstrate unique metabolic pathways that contain both photosynthetic and heterotrophic processes, exhibiting their great potential for future biotechnological applications, especially in addressing global challenges related to ecosystem restoration. In addition, their diverse reproductive strategies and life cycles, which predominantly include asexual reproduction, reflect their evolutionary adaptations, enabling them to thrive and survive in various environmental conditions. Over time, classification strategies have considerably enhanced, showing promising advances in microalgae taxonomy, moving from morphological approaches to molecular biology techniques. This progression has revealed the extensive genetic diversity within microalgae. Research studies are essential for understanding the ecological importance of microalgal genetics as well as for exploring their untapped potential in commercial and scientific applications, ranging from biofuels to pharmaceuticals.

Nutritional food sources remain limited in the face of a rapidly growing population, and at this point, microalgae are evaluated as nutritious and functional resources. With their rich content, microalgae enhance the composition of food products and facilitate their functionalization. In this context, the nutritional value and the rheological and sensory properties of products can improve through the addition of microalgae. A correlation has been observed between the amount of microalgal biomass added and the overall acceptability of the product. Additionally, the intense pigment content of microalgae can significantly influence the color, taste, and quality of the product. Furthermore, studies have shown that the functional properties of microalgae may help reduce the risk of certain health issues.

In summary, the versatile properties of microalgae, ranging from their rich nutritional content to their diverse biological activities and environmental benefits, position them as valuable assets in addressing various global challenges related to food and health. The substantial nutritional value present in various microalgae species has garnered considerable attention from the food industry, with expectations for even greater interest in the future.

## Figures and Tables

**Table 1:** Several studies underscore the biological features of microalgae species.

Biological Features	Microalgae Species	Outcomes	References
Photosynthesis	*Chlorella vulgaris* (Chlorophyta)	Enhance CO_2_ fixation efficiency	[2]
	Phytoplankton	Small phytoplankton flourish in changing climates	[33]
	*Skeletonema marinoi* (formerly *Skeletonema costatum)* (Mediophyceae)	Increase extracellular carbonic anhydrase (eCA) activity	[34]
	*Phaeocystis globose* (Prymnesiophyceae)	Stable internal carbonic anhydrase (iCA) activity	
	*Gephyrocapsa huxleyi* (formerly *Emiliania huxleyi*) (Coccolithophyceae)	Low eCA activity	
	*Dunaliella salina* (Chlorophyta)	Salinity stress enhanced microalgae photosynthesis and pigment production	[35]
	*Desmodesmus abundans* (formerly *Chlorella fusca*) (Chlorophyta)	Layer thickness optimizes microalgae photosynthesis in thin-layer cascade systems	[36]
	*Scenedesmus almeriensis* (Chlorophyta)	Optimize photosynthesis and cost are key for microalgal biofuel	[4]
Cellular structure	*Phaeodactylum tricornutum* (Bacillariophyceae)	Enhance diatom photosynthesis efficiency	[37]
		Environmental stress influences microalgae cell structure and morphology	[6]
	*Dunaliella* spp. (Chlorophyta)	Genetic analysis reveals diverse cell structures in *Dunaliella* strains	[38]
	*Phaeodactylum tricornutum* (Bacillariophyceae) *Skeletonema* sp. (Mediophyceae) *Porphyridium* sp. (Rhodophyta) *Tetraselmis striata* (Chlorophyta)	Microalgal cell structures produce bioactive compounds with therapeutic potential	[39]
	*Chlorella* spp. (Chlorophyta)	Its cell structures optimize lipid production for biodiesel	[40]
	*Chlorella vulgaris* (Chlorophyta)	Pressure-assisted ozonation method is more efficient than ultrasonication in *Chlorella vulgaris* cell disruption	[41]
		Stress conditions promote lipid accumulation in *Chlorella* cell structures	[42]
Metabolism	*Diacronema lutheri* (formerly *Pavlova lutheri*) (Pavlovophyceae) *Gephyrocapsa huxleyi* (formerly *Emiliania huxleyi*) (Coccolithophyceae) *Cyanophora paradoxa* (Glaucophyta)	Plastid glucose-6-phosphate isomerase is essential for primary metabolism in microalgae	[43]
	*Chlamydomonas reinhardtii* (Chlorophyta)	Demonstrate varied metabolic efficiency across growth conditions	[10]
	*Galdieria sulphuraria* (Cyanidiophyceae)	Exhibit metabolic flexibility through unique genes	[44]
		Enhance metabolic versatility via sugar uptake pathway	[45]
		Metabolize diverse carbon sources heterotrophically	[46]
		Metabolize various sugars for heterotrophic growth	[47]
	*Chlorella vulgaris* (Chlorophyta)	Exhibit efficient hexose transport and regulation	[48]
		Enhance nutrient uptake for efficient metabolism	[49]
		Microalgae growth affects nitrogen uptake efficiency in *Chlorella vulgaris*	[50]
	*Chlorella sorokiniana* (Chlorophyta) *Chlorella vulgaris* (Chlorophyta)	Bacterial co-immobilization enhances lipid metabolism in microalgae	[51]
	*Phaeodactylum tricornutum* (Bacillariophyceae)	Glycerol enhances biomass and lipid metabolism in diatom *Phaeodactylum*	[52]
	*Rhizomonas salina* (formerly *Pyrenomonas salina)* (Cryptophyceae)	Nitrogen depletion reduces phycoerythrin, affecting cryptophyte metabolism	[53]
Reproduction and life cycle	*Symbiodinium* (Dinophyceae) strains	*Symbiodinium* reproduction varies by strain, influenced by growth and stress	[54]
	*Chlorella* spp. (Chlorophyta)	Microplastics inhibit microalgae growth and reproduction	[14]
	*Tetraselmis suecica* (Chlorophyta) *Desmodesmus armatus* (formerly *Scenedesmus armatus)* (Chlorophyta) *Microchloropsis gaditana* (formerly *Nannochloropsis gaditana)* (Eustigmatophyceae)	Microplastics reduce biomass and alter microalgae cell growth	[55]
Diversity and classification	Euphotic zone samples	Exhibit vast, uncultured diversity across varying ocean depths	[15]
	*Prasiolale* species	Exhibit distinct clades with varying diversity across habitats	[56]
	*Scenedesmus obliquus* *Chlorella sorokiniana* and others.	Exhibit diverse classifications, expanding biofuel production potential	[57]

**Table 2:** Compositional data for main commerical species.

Commerical Species	Biomass Dry Wight (%)			References
	Protein	Carbohydrate	Lipid	
*Limnospira platensis* (formerly *Spirulina platensis*)	60–70%	15–20%	5–8%	[31]
*Chlorella vulgaris*	42–58%	12–55%	5–40%	[79]
*Dunaliella salina*	19-57%	5.6–40%	18-43%	[146,148,149,150]
*Haematococcus lacustris* (formerly *Haematococcus pluvialis*)	29-45%	15–63%	20-25%	[147]

**Table 3:** The use of microalgae in food products.

Product Types	Microalgae	Outcome	References
Soymilk vegan kefir	*Haematococcus lacustris* (Chlorophyta)	-Improve protein content -Improve bacterial diversity	[154]
Yogurt drink	*Limnospira platensis* (formerly *Spirulina platensis*) (*Cyanobacteria*)	-Enhance sensory and rheological properties	[153]
Sauce		-Improve bacterial viability	[155]
Ice cream		-Enhance sensory and rheological properties	[156]
Wheat tortillas	*Nannochloropsis* sp. (Eustigmatophyceae) *Tetraselmis* sp. (Chlorophyta)	-Enhance color properties -Improve protein, lipid, and ash contents	[20]
Pasta	*Limnospira platensis* (formerly *Spirulina platensis*) (*Cyanobacteria*)	-Enhance color properties -Improve protein and mineral contents -Influence sensory acceptance	[152]
Wheat bread	*Tetraselmis chui* (Chlorophyta)	-Improve protein content -Enhance color properties	[157]
Bread	*Chlamydomonas* sp. *Nannochloropsis gaditana* (Eustigmatophyceae)	-Enhance color properties -Improve protein and ash contents	[158]
Ice cream	*Nannochloropsis oculata* (Eustigmatophyceae) *Diacronema vlkianum* (Pavlovophyceae) *Porphyridium purpureum* (Rhodophyta)	-Enhance color properties	[22]
